# Impact of Anabolic–Androgenic Steroid Abuse on the Cardiovascular System: Molecular Mechanisms and Clinical Implications

**DOI:** 10.3390/ijms262211037

**Published:** 2025-11-14

**Authors:** Antoni Borowiec, Iga Waluszewska, Michał Jurkiewicz, Wioletta Szczurek-Wasilewicz

**Affiliations:** 1 Students’ Scientific Society, Department of Pharmacology, Faculty of Medicine, University of Opole, 45-052 Opole, Poland; 2 Students’ Scientific Society, 3rd Department of Cardiology, Faculty of Medical Sciences in Zabrze, Medical University of Silesia, 40-055 Katowice, Poland; 3 Department of Pharmacology, Faculty of Medicine, University of Opole, 45-052 Opole, Poland; 4 2nd Department of Cardiology and Angiology, Silesian Center for Heart Diseases, 41-800 Zabrze, Poland

**Keywords:** heart failure, anabolic–androgenic steroid, cardiovascular system, risk factors

## Abstract

Anabolic–androgenic steroids (AAS) are synthetic derivatives of testosterone that are used therapeutically but are frequently abused by athletes and individuals seeking to increase muscle mass. Their anabolic (promoting muscle growth) and androgenic (inducing masculine characteristics) effects result from androgen receptor activation in target tissues. However, chronic supraphysiological AAS exposure is associated with serious cardiovascular consequences, ranging from hypertension and lipid disorders to cardiomyopathy, atherosclerosis, and sudden cardiac death. This review provides an updated and integrative perspective on both the molecular and clinical aspects of AAS-induced cardiovascular toxicity, highlighting recent advances in understanding endothelial injury, oxidative stress, fibrosis, and arrhythmogenesis. Importantly, it emphasizes the emerging recognition of AAS abuse as a modifiable cardiovascular risk factor and discusses potential preventive and therapeutic strategies, including early cardiovascular screening and risk stratification. Understanding these mechanisms is essential for recognizing the clinical manifestations of AAS misuse and for improving cardiovascular risk assessment in affected individuals. These insights underscore the clinical significance of AAS abuse as a cardiovascular risk factor and the need for vigilant cardiac monitoring and early intervention in this population.

## 1. Introduction

Anabolic–androgenic steroids (AAS) are a class of hormones including testosterone and synthetic analogs, characterized by anabolic and androgenic properties. Androgens interact with androgen receptors, triggering the development of male primary and secondary sexual characteristics, along with various systemic effects [1]. Clinically, certain AAS are prescribed at physiological doses for conditions such as male hypogonadism, muscle wasting diseases, and certain anemias. In contrast, non-medical AAS use typically involves supraphysiological dosages and polypharmacy “cycles”, intended to boost muscle mass and athletic performance [2,3,4,5]. Since their introduction in sports in the mid-20th centure, AAS abuse has become an epidemic among recreational bodybuilders and athletes, predominantly young men [5,6,7]. Global prevalence estimates suggest that about 6% of men and 1–2% of women worldwide have used AAS at least once in their lifetime, with the highest prevalence observed in Europe, the Middle East, and South America [4,8,9]. In specific high-risk populations—such as recreational bodybuilders, soldiers, and prisoners—this percentage may exceed 30–50%. In Western countries, lifetime prevalence is estimated at 1–5%, with over 90% of users being male and typically 18–35 years old [8,9,10]. Notably, AAS use among women and older adults is rising, and new “designer” steroids are increasingly available online [8,10].

Commonly abused AAS include injectable testosterone esters (enanthate, cypionate, propionate), plus synthetics like nandrolone decanoate, stanozolol, methandrostenolone, trenbolone, oxandrolone, and boldenone. Abusers often “stack” multiple steroids or “cycle” them to maximize gains and minimize side effects, taking doses 5–20 times higher than therapeutic recommendations [9,10,11,12]. Many users also take ancillary drugs (growth hormone, insulin, thyroid hormone, clenbuterol, etc.) to amplify anabolic effects or reduce side effects, which can further complicate cardiovascular risk [12,13]. Over one-third of chronic users develop AAS dependence despite adverse consequences [5].

Many studies have linked chronic AAS abuse to a spectrum of cardiovascular complications, initially noted in case reports of young users presenting with unexpected myocardial infarction, stroke, or sudden cardiac death (SCD) [6,7]. Subsequent clinical studies and reviews have confirmed associations between long-term AAS use and increased incidence of hypertension, premature coronary artery disease, cardiomyopathy, arrhythmias, thromboembolic events, and heart failure [1,3,5,6]. From the pathological point of view, supratherapeutic androgen exposure affects the cardiovascular system both directly (via cardiac and vascular androgen receptors) and indirectly (via lipid abnormalities, coagulation changes, and neurohormonal shifts), ultimately promoting atherosclerosis, myocardial fibrosis, and electrical instability. [1,2,3,4]. This review summarizes the current knowledge about the cardiovascular effects of AAS, with particular emphasis on molecular mechanisms and clinical implications.

## 2. Epidemiology of AAS Use

AAS abuse became prevalent in the general population by the 1980s and has since grown into a major problem. In most Western countries, lifetime AAS use among the general population is estimated at 1–5%, with significantly higher prevalence in some age groups [4,8,9]. A study by Pope et al. estimated that 2.9–4.0 million Americans aged 13–50 (roughly 1% of this demographic) have used AAS at some point in their lives [4]. Over 90% of users are male, typically aged between late teens and 30, although the percentage of women and older people is also non-negligible. Among high-risk groups include recreational weightlifters and bodybuilders (in some gyms this percentage is 30–50%), strength athletes, soldiers, and even prisoners [8,10]. It should be noted that epidemiological data on AAS use have important limitations: self-reported surveys are prone to underreporting and varying definitions, so the true prevalence of abuse is likely higher. Additionally, differences in study methodologies and social stigma likely lead to further underestimation of use.

## 3. Molecular Mechanisms of AAS Cardiovascular Toxicity

**Figure 1:**
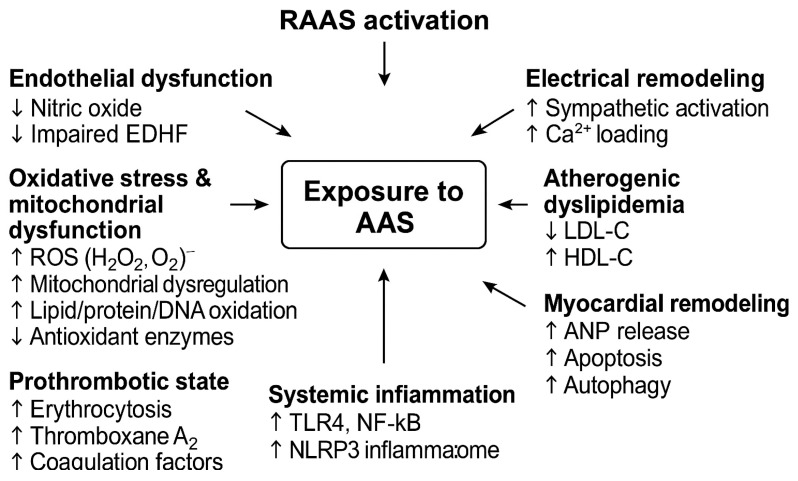
Baseline pathophysiological mechanisms of anabolic–androgenic steroids (AAS) on the cardiovascular system. Abbreviations: AAS, anabolic-androgenic steroids; ANP, atrial natriuretic peptide; DNA, deoxyribonucleic acid; EDHF, endothelium-derived hyperpolarizing factor; HDL-C, high-density lipoprotein cholesterol; LDL-C, low-density lipoprotein cholesterol; NF-κB, nuclear factor-κB; NLRP3, NOD-like receptor family pyrin domain containing 3; RAAS, renin–angiotensin–aldosterone system; ROS, reactive oxygen species; TLR4, Toll-like receptor 4. ↑—increase, ↓—decrease.

Chronic exposure to supraphysiologic doses of AAS induces a variety of pathophysiological changes in the cardiovascular system. The mechanisms are multifactorial, involving direct androgen receptor-mediated effects on cardiac and vascular tissues as well as indirect metabolic and hemodynamic effects. Key pathways include endothelial dysfunction, adverse lipid profile changes, prothrombotic and vasospastic effects, myocardial hypertrophy with fibrosis, and electrical remodeling contributing to arrhythmias. The pathophysiological mechanisms of AAS and their potential cardiovascular consequences are presented in Table 1 and Figure 1.

This schematic diagram illustrates how AAS abuse triggers androgen receptor-mediated hypertrophy, endothelial dysfunction, oxidative stress, and inflammation, ultimately leading to myocardial fibrosis, arrhythmias, and increased cardiovascular risk.

### 3.1. Effect of AAS on Androgen Receptors in the Heart and Endothelium

The molecular cardiac effects of AAS are initiated by their binding to androgen receptor (AR) expressed in cardiomyocytes. Both testosterone and dihydrotestosterone (DHT) activate AR, promoting protein synthesis within cardiac myocytes and stimulating the release of atrial natriuretic peptide (ANP), which induces a direct hypertrophic response [14,15,16]. Furthermore, AR stimulation increases the expression and number of androgen receptors in the myocardium, and chronic abuse of AAS leads to changes in cardiac phenotype and the induction of ventricular hypertrophy through genomic and non-genomic signaling mechanisms [8,17,18]. Importantly, AAS not only activate existing receptors but also induce profound metabolic and structural changes that may impair both the electrical and mechanical properties of the myocardium [17,18]. Additionally, AR are found in cardiomyocytes as well as in vascular endothelial and smooth-muscle cells, where they regulate nitric oxide (NO) synthesis and vascular tone. Under physiological conditions, activation of endothelial AR by testosterone or DHT may have a protective effect by increasing nitric oxide (NO) synthesis in endothelial cells via the mitogen-activated protein kinase (MAPK) and phosphatidylinositol 3-kinase (PI3K)/protein kinase B (AKT) pathways [14,15,16,17,18]. Furthermore, activation of AR in the vascular endothelium may increase prostacyclin (PGI_2_) production by inducing cyclooxygenase-2 and enhance the release of endothelium-derived hyperpolarizing factor (EDHF), which together promote vasodilation [18,19]. However, excessive AR stimulation by supraphysiological AAS doses disrupts endothelial homeostasis, leading to decreased NO bioavailability, increased oxidative stress, and RAAS overactivation [15,16,17,18,19].

High androgen concentrations enhance the proinflammatory response in the endothelium by increasing the expression of adhesion molecules: Vascular cell adhesion molecule-1 (VCAM-1), Intercellular adhesion molecule-1 (ICAM-1), and E-selectin and by increasing monocyte adhesion to the endothelium [20,21]. In turn, under conditions of moderate AR stimulation, the opposite effect is possible, contributing to the inhibition of nuclear factor kappa B (NF-Κb) pathways and reduced expression of adhesion molecules [21,22]. However, during AAS abuse, the harmful effects predominate. In summary, AAS abuse directly affects both cardiac and vascular cells through androgen receptor-mediated pathways, initiating a cascade of molecular events that culminate in cardiac hypertrophy and endothelial dysfunction [23,24,25,26].

### 3.2. Effects of AAS on Oxidative Stress and Mitochondrial Dysfunction

Recent evidence further supports oxidative stress as a key mechanism of AAS cardiotoxicity [25,26]. Supraphysiological doses of AAS significantly disrupt redox balance in cardiomyocytes [23]. Experimental studies have shown that high doses of nandrolone or testosterone significantly increase hydrogen peroxide (H_2_O_2_) production in cardiac tissue. Moreover, nandrolone inhibits the physiological influx of Ca^2+^ ions into cardiomyocyte mitochondria and reduces mitochondrial membrane potential, being early substrates for myocardial injury [23,24]. Consequently, mitochondrial function is impaired. Both nandrolone and testosterone promote the accumulation of reactive oxygen species (ROS) in the myocardium, leading to lipid and protein oxidation, as evidenced by increased levels of malondialdehyde, 8-hydroxyguanosine (8-OHdG), and protein carbonylation in cardiac tissue [24,25]. Simultaneously, AAS reduce the activity of antioxidant enzymes, which shifts the oxidant-antioxidant balance toward a pro-oxidant state. These disturbances are accompanied by activation of the molecular cascade of innate immunity, including increased expression of toll-like receptors (TLR4), NF-κB, and the nucleotide oligomerization domain (NOD)-like receptor family pyrin domain containing 3 (NLRP3) inflammasome [25,26]. Thus, oxidative stress induced by AAS triggers an inflammatory response in the heart, further exacerbating cellular damage. Furthermore, renin–angiotensin–aldosterone system (RAAS) dysregulation with elevated aldosterone levels in chronic AAS users may further contribute to the generation of oxidative stress [27]. In turn, aldosterone, via activation of the mineralocorticoid receptor, promotes myocardial ROS generation, inflammation, and fibrosis, which leads to myocardial remodeling. The cumulative oxidative damage and progressive mitochondrial dysfunction form a vicious cycle—impaired mitochondria produce excess ROS, which in turn further deteriorate mitochondrial integrity [23,27,28]. This mechanism underlies long-term cardiac complications seen in chronic AAS users. Moreover, sympathetic overactivity described in AAS users may further potentiate oxidative and inflammatory responses, contributing to autonomic imbalance and arrhythmogenesis [29]. Over time, these pathological processes may culminate in structural myocardial alterations such as left ventricular hypertrophy, interstitial fibrosis, and impaired diastolic function. Importantly, antioxidant interventions may offer partial cardioprotection in AAS users [25,30]. In experimental model, administration of N-acetylcysteine to rats treated with nandrolone resulted in decreased levels of 8-OHdG, reduced activation of TLR4/NF-κB/NLRP3 pathways, which also contributed to reduced myocardial damage. [25]. These findings underscore oxidative stress as a one of the central mediators of AAS-induced cardiotoxicity, suggesting that mitigating oxidative damage may help protect the heart against the long-term consequences of steroid abuse.

### 3.3. Effects of ASS on Apoptosis and Autophagy

Excessive AR stimulation and oxidative stress can trigger programmed cell death of cardiomyocytes via apoptosis. More than two decades ago, researchers discovered that several commonly abused AAS (e.g., stanozolol, high-dose testosterone esters) can induce cardiomyocyte apoptosis in a dose-dependent manner [31,32]. These findings provided the first direct evidence that anabolic steroid abuse may initiate programmed death of cardiac cells, contributing to the loss of viable myocytes, progressive myocardial thinning, pathological remodeling, cardiomyopathy, and ultimately, an increased risk of sudden cardiac death [31,32,33]. Subsequent in vivo studies confirmed the pro-apoptotic properties of AAS. A significantly higher number of apoptotic cells (TUNEL-positive) was observed in the hearts of rats chronically exposed to nandrolone compared to control animals [25]. On a molecular level, AAS activate the intrinsic pathway of mitochondrial apoptosis: they increase the release of cytochrome c from mitochondria into the cytoplasm upon membrane damage in cardiomyocytes, elevate the ratio of caspase-3 in its activated form to the proenzyme, and induce the expression of the pro-apoptotic factor p53 [25,34]. Simultaneously, AAS have been found to enhance inflammatory pathways (TLR4/NF-κB), which may further promote cell death through inflammatory apoptosis dependent on the NLRP3 inflammasome [25]. These results indicate that anabolic steroid abuse triggers programmed cell death processes in the heart. The effect of AAS on autophagy is less well recognized [35,36], but it is likely that long-term exposure to steroids also disrupts this homeostatic mechanism. Hypertrophic and anabolic signals activated by AAS (e.g., via the Akt/mammalian target of rapamycin (mTOR) pathway) may inhibit autophagy in cardiomyocytes, similarly to other models of cardiac hypertrophy unrelated to AAS [37,38,39]. Impaired autophagy contributes to impaired clearance of damaged mitochondria and protein aggregates, promoting further cell damage and the development of cardiac dysfunction [35,36,37]. Although conclusive experimental data on autophagy in the context of AAS are lacking, numerous pathological phenomena observed in the hearts of steroid users such as the accumulation of abnormal structures in cardiomyocytes may result from autophagy disorders [40]. It is worth emphasizing the need for further research on this aspect, as the balance between autophagy and apoptosis is crucial for cardiac cell survival during stress.

## 4. Myocardial Remodeling and Fibrosis in AAS Users

A consequence of AAS-induced pathophysiological processes is progressive myocardial remodeling, including concentric hypertrophy, altered geometry, and interstitial fibrosis. Echocardiography and cardiac magnetic resonance (CMR) imaging in steroids-abusing athletes have demonstrated increased septal and posterior wall thickness and ventricular dilation compared with non-users [41,42]. Furthermore, functional assessment frequently reveals reduced left ventricular systolic performance, including diminished peak myocardial strain, and early diastolic dysfunction characterized by a reduced E/A ratio, reflecting impaired relaxation secondary to increased wall stiffness [40,41,43]. Histopathological evidence from heart biopsies and autopsies of AAS users show diffuse interstitial fibrosis and focal myocyte necrosis [41,44]. Furthermore, myocardial fibrosis induced by AAS is fueled by chronic subclinical inflammation, which stimulates fibroblasts to produce extracellular matrix. Over time, depletion of compensatory mechanisms leads to ventricular dilation and clinical heart failure [40]. The clinical manifestation of heart failure in AAS users is often described as “steroid cardiomyopathy”. Case reports describe instances of severe dilated cardiomyopathy and advanced heart failure in bodybuilders between 30 and 40 years of age, often with persistent and irreversible ventricular dysfunction despite the discontinuation of AAS [41,42,43]. Chronic AAS abuse thus promotes a mixed hypertrophic–fibrosing phenotype of cardiomyopathy, substantially increasing the risk of premature heart failure and sudden cardiac death [42,43].

## 5. Effects of Anabolic–Androgenic Steroids on the Coagulation System

Abuse of AAS induces profound alterations in hemostatic balance, shifting it toward a prothrombotic state [45,46]. Numerous case reports demonstrated that young AAS users without significant atherosclerosis or conventional cardiovascular risk factors experience acute thromboembolic events, including myocardial infarction, ischemic stroke, deep vein thrombosis, and pulmonary embolism [47,48,49]. The pathophysiology underlying this phenomenon is multifactorial and involves erythropoietic stimulation, platelet hyperreactivity, increased synthesis of coagulation factors, and impaired fibrinolysis [47,48,49,50,51]. AAS promote erythropoiesis via a dual mechanism comprising an early, transient stimulation of erythropoietin and a sustained suppression of hepcidin, thereby increasing iron availability; this leads to rises in hemoglobin/hematocrit and, consequently, blood viscosity. In parallel, androgens directly modulate platelet function by upregulating thromboxane A_2_ (TXA_2_) receptor density on platelet membranes and enhancing TXA_2_-mediated signaling and aggregation in response to activating stimuli [52,53]. The study by Ajayi et al. demonstrated that supraphysiological testosterone administration significantly increased platelet aggregation and upregulated TXA_2_ receptor density on platelet membranes, resulting in enhanced TXA_2_-mediated responses, as reflected by increased thromboxane B_2_ (TXB_2_) release upon activation [53]. The effect of AAS on hepatic synthesis of coagulation factors remains inconsistent. Some studies have reported increased plasma levels of factors II, VIII, and IX, suggesting a procoagulant shift [45,46], whereas other investigations did not confirm these findings or demonstrated only transient alterations [50,54]. Data regarding factors VII, X, and fibrinogen are similarly inconclusive [55,56], indicating that the overall impact of AAS on coagulation protein synthesis is heterogeneous and not yet fully understood. AAS also affect the fibrinolytic system. Sidelmann et al. reported reduced fibrinolytic activity in individuals using AAS, despite no significant changes in thrombin activatable fibrinolysis inhibitor (TAFI) and plasminogen activator inhibitor type 1 (PAI-1) concentrations [51]. Furthermore, this effect is likely not due to changes in fibrin structure but rather to increased plasma fibrinogen, factor XIII, and plasmin inhibitor concentrations [57]. Endothelial dysfunction likely contributes to fibrinolytic abnormalities in AAS users [8,16]. Clinically, chronic AAS use is also associated with endothelial dysfunction, evidenced by reduced endothelium-dependent vasodilation (FMD). Paradoxically, endothelial injury caused by AAS may transiently elevate circulating tissue plasminogen activator (tPA); however, PAI-1—often present in relative molar excess—rapidly forms inactive tPA–PAI-1 complexes, thereby limiting effective clot lysis [8,57]. Collectively, the hemostatic phenotype in AAS users is modulated by the specific compound, cumulative dose, way of administration, and duration of exposure. Importantly, a proportion of coagulation-system abnormalities appears at least partially reversible following cessation of AAS.

## 6. Effect of AAS on Lipid Metabolism and Blood Pressure Regulation

The effect of AAS on lipid metabolism and blood pressure is well documented. AAS induce an atherogenic lipid profile that significantly increases the risk of atherosclerosis [27,58]. Even short-term steroid cycles (lasting several weeks) can reduce high-density lipoprotein (HDL) cholesterol level by 20–70% and concomitantly raise low-density lipoprotein (LDL) cholesterol by approximately 20%, often with a moderate rise in total cholesterol [59,60]. AAS affect the lipid profile by modifying key hepatic cholesterol metabolism pathways. They suppress apolipoprotein A-I expression and enzymes involved in HDL production, while upregulating hepatic very-low-density lipoprotein (VLDL)/LDL synthesis [59,60,61]. Moreover, AAS also accelerate HDL catabolism, further exacerbating dyslipidemia. These adverse lipid changes, combined with AAS-induced endothelial dysfunction, lead to accelerated atherosclerosis in AAS users [28,58,60]. Baggish et al. reported that AAS users exhibited significantly greater coronary plaque volume than non-users, and that cumulative AAS exposure was positively correlated with atherosclerosis severity [62]. They support the concept of a cumulative effect, whereby prolonged or high-dose AAS use is associated with earlier onset and greater severity of coronary artery disease [62].

Anabolic–androgenic steroids also affect blood pressure regulation through effects on the RAAS and sympathetic nervous system. AAS can increase sympathetic activity both directly by increasing sympathetic tone and indirectly by stimulating renin release in the kidneys [28,63]. Furthermore, chronic AAS use dysregulates the RAAS by stimulating excess aldosterone secretion [27]. In turn, aldosterone acts on the kidneys, leading to sodium and water retention and exerts direct harmful effects on the cardiovascular system, promoting oxidative stress and fibrosis [27,28]. AAS may also upregulate angiotensin II type 1 receptors in cardiac and vascular tissues, potentially enhancing androgen-dependent VCAM-1 expression via AR/Angiotensin II type 1 receptor (AT1R)/NF-κB signaling [27,64,65]. Additionally, AAS impair vasodilatory control by reducing endothelial release of NO and prostacyclin, which physiologically cause vasodilation and oppose blood pressure elevation [28,66]. Moreover, AAS users do not experience the typical post-exercise hypotension, suggesting persistently elevated sympathetic activation and impaired vascular reflex regulation [66]. Chronic adrenergic stimulation and excessive activation of the RAA by AAS contribute to cardiac pressure overload, the development of pathological cardiac hypertrophy, endothelial dysfunction, and myocardial fibrosis [27,28,63]. Changes caused by AAS are not always reversible, and high blood pressure and dyslipidemia may persist in some users even after AAS discontinuation [27,28].

## 7. Other Neurohormonal Effects of AAS Use and Their Cardiovascular Implications

The use of AAS disrupts the physiological balance of several neuroendocrine axes, causing systemic effects that extend to the cardiovascular system. Exogenous androgens strongly suppress the hypothalamic–pituitary–gonadal axis through negative feedback, reducing luteinizing hormone (LH) and follicle-stimulating hormone (FSH) secretion. This in turn leads to decreased endogenous testosterone synthesis and impaired spermatogenesis [67,68]. At the molecular level, exogenous androgens suppress the pulsatile secretion of gonadotropin-releasing hormone (GnRH) from the hypothalamus and reduce pituitary responsiveness to GnRH, thereby lowering LH and FSH secretion. In Leydig cells, reduced LH signaling results in diminished cyclic adenosine 3′,5′-monophosphate (cAMP)–protein kinase A (PKA) activity and downregulation of key steroidogenic enzymes, which impairs testosterone biosynthesis. In parallel, reduced FSH levels and lower intratesticular testosterone weaken the FSH/cAMP–PKA–dependent support of Sertoli cell function, disrupting spermatogenesis [68,69,70,71]. Clinically, these effects are reflected by testicular atrophy, infertility, and hypogonadism observed after AAS discontinuation [68]. From a cardiovascular standpoint, hypogonadism is associated with endothelial dysfunction, increased visceral adiposity, insulin resistance, and an adverse metabolic profile, all of which contribute to accelerated atherosclerosis and increased cardiovascular risk [10].

Anabolic–androgenic steroids also modulate the activity of the hypothalamic-pituitary-thyroid axis [67]. Long-term AAS use can reduce total thyroid hormone concentrations by decreasing thyroxine-binding globulin (TBG), typically without lowering free triiodothyronine (T3)/thyroxine (T4) hormone levels [72,73]. Reports of transient thyroid-stimulating hormone (TSH) fluctuations during or after AAS cycles exist, but their direction and magnitude are inconsistent [72,73,74]. This likely reflects a modest and variable pituitary response to changes in thyroid hormone availability. Prolonged suppression of thyroid hormones may lead to symptoms of subclinical hypothyroidism, which is a state linked with dyslipidemia, impaired myocardial relaxation, increased systemic vascular resistance, and a greater risk of coronary artery disease [73,74]. In contrast, the influence of AAS on the hypothalamic–pituitary–adrenal axis appears to be limited [67,74]. Even supraphysiological doses of testosterone do not substantially alter adrenocorticotropic hormone (ACTH) or cortisol levels, indicating that AAS do not significantly suppress adrenal function in most cases [67].

Altogether, neurohormonal alterations induced by AAS, including gonadal suppression and thyroid imbalance, act synergistically with direct metabolic and vascular effects of steroids. These disturbances aggravate dyslipidemia, vascular stiffness, and insulin resistance, thereby accelerating atherosclerosis, promoting left ventricular hypertrophy, and increasing susceptibility to arrhythmias and sudden cardiac death [62,67,74]. In this way, neuroendocrine disruption constitutes a crucial and often underestimated component of the overall cardiovascular toxicity of AAS.

## 8. Arrhythmias and Electrical Remodeling in AAS Users

Growing evidence indicates that AAS overuse increases susceptibility to supraventricular and ventricular arrhythmias and may lead to sudden cardiac death. Many studies have confirmed that steroid users frequently report palpitations and exhibit premature ventricular beats, non-sustained ventricular tachycardia, and episodes of atrial fibrillation [40,41,42,43]. The underlying cause of these arrhythmias in AAS users is believed to involve autonomic nervous system dysfunction. AAS users demonstrate reduced heart-rate variability and a shift toward sympathetic dominance, consistent with heightened adrenergic tone and impaired vagal modulation [29]. Moreover, AAS users often do not manifest the typical post-exercise hypotension observed in non-users, suggesting persistent sympathetic activation and abnormal vascular reflex control that together may lower the threshold for arrhythmias [66]. Chronic sympathetic predominance can facilitate both triggered activity and reentry by increasing dispersion of repolarization and intracellular calcium loading, thereby priming myocardium for afterdepolarizations. Furthermore, structural remodeling provides additional substrate for arrhythmias AAS exposure is associated with concentric hypertrophy, chamber dilation, and diffuse interstitial fibrosis as observed on echocardiography and CMR imaging. Long-term follow-up studies have shown that former users can exhibit persistent alterations in cardiac structure and function despite discontinuing steroids [41,42,43]. Fibrosis and myocyte disarray slow conduction and increase spatial heterogeneity of refractoriness, supporting macro- and micro-reentrant circuits. In some reports, advanced CMR techniques (late gadolinium enhancement and abnormal T1/T2 mapping indices) suggest a fibrotic-inflammatory myocardial phenotype in AAS users, which plausibly augments arrhythmic risk beyond hemodynamic effects alone [41,42,43]. At the cellular level, supraphysiologic androgens promote oxidative stress, mitochondrial dysfunction, and calcium mishandling in cardiomyocytes [23,24,25]. Elevated ROS and mitochondrial membrane depolarization destabilize action-potential morphology and facilitate early and delayed afterdepolarizations. In parallel, androgen-induced activation of innate immune pathways (TLR4/NF-κB/NLRP3) and mitochondrial cytochrome-c release promote apoptosis. This loss of electrically coupled myocytes thins the viable myocardium and further amplifies conduction heterogeneity [25]. Although the direct effects of supraphysiologic androgens on specific ion channels in the human heart remain incompletely characterized, animal and in vitro studies indicate that oxidative and inflammatory stress can alter L-type Ca^2+^ currents and repolarizing K^+^ currents. These changes favor triggered activity and prolong repolarization in vulnerable regions of the myocardium [23,24,25]. Some experimental models have confirmed increased arrhythmia susceptibility due to AAS [31,34,65]. Administration of anabolic steroids during adolescence induces long-term cardiac hypertrophy and increases vulnerability to ischemia/reperfusion-related ventricular arrhythmias in adult rats, implying durable pro-arrhythmic remodeling after exposure during critical developmental windows [65]. Additionally, in vitro studies demonstrate that testosterone and stanozolol have pro-apoptotic effects on cardiomyocytes [31,34], whereas nandrolone triggers ROS-mediated cellular injury [23,24,25]. These effects converge on a common arrhythmogenic pathway characterized by the loss of electrically coupled myocytes, patchy fibrosis, and calcium/ROS-driven afterdepolarizations.

Finally, these mechanisms interact with other AAS-related cardiovascular perturbations. AAS-induced hypertension and activation of RAAS increase left ventricular wall stress and promote fibrosis, while excess aldosterone further augments ROS production and collagen deposition [27,28,66]. Concurrent dyslipidemia and accelerated atherosclrosis may introduce ischemic triggers, and a prothrombotic milieu increases the risk of acute coronary events that can precipitate malignant ventricular arrhythmias [45,46,47,48,49,50,51,52,53,58,59,60,61,62]. In individuals abusing AAS who suffered sudden cardiac death, toxicological analyses most frequently revealed nandrolone, testosterone, and stanozolol. The predominant macroscopic findings were cardiomegaly and left ventricular hypertrophy, while histology showed myocardial fibrosis and necrosis. Four main mechanisms appear to underlie arrhythmias and sudden cardiac death in AAS users: the atherogenic, thrombosis, vasospasm (due to impaired nitric oxide signaling), and direct myocardial injury models. Hypertrophy, fibrosis, and necrosis create an arrhythmogenic substrate, and AAS use transforms physiological remodeling in athletes into pathological hypertrophy, increasing the risk of fatal arrhythmias [40].

To underscore the clinical impact of AAS abuse, Table 2 provides an overview of major clinical studies investigating the cardiovascular outcomes associated with chronic AAS use.

## 9. Conclusions

Abuse of AAS at high doses exerts negative effects on the cardiovascular system. Through a combination of endothelial damage, adverse metabolic changes, and direct toxic effects on the myocardium, the effects of AAS provide a substrate for premature cardiovascular disease. Numerous studies indicate that inappropriate AAS use can lead to accelerated coronary atherosclerosis, thrombosis, hypertension, pathological cardiac hypertrophy, and electrical instability, which consequently contribute to myocardial infarction, heart failure, arrhythmias, and sudden cardiac death. Due to society’s changing lifestyle, AAS abuse is an emerging cardiovascular risk factor that clinicians should consider in the differential diagnosis of cardiovascular disease. Unfortunately, many cases of AAS-related cardiomyopathy or myocardial infarction may remain undiagnosed if the patient’s history of drug use is not properly assessed. Therefore, cardiologists and primary care physicians should consider the use of AAS in young patients with unexplained left ventricular dysfunction, early coronary artery disease, or arrhythmias, especially if there are physical or laboratory findings. In addition, AAS users should undergo cardiovascular risk evaluation, including blood pressure monitoring, lipid panels, and possibly echocardiography. Studies in the last decade have demonstrated the mechanisms how AAS affect the cardiovascular system—dyslipidemia, endothelial dysfunction, prothrombotic changes, myocardial fibrosis, and arrhythmogenesis—and confirmed long-suspected clinical links with heart disease However, the long-term cardiovascular prognosis of former AAS users remains uncertain, as some steroid-induced changes may persist even after cessation. Future research should focus on quantifying the dose-dependent risk threshold for irreversible cardiovascular damage, developing strategies for early detection of AAS-induced cardiac injury, and implementing effective educational and rehabilitation programs to reduce AAS use in society.

## Figures and Tables

**Table 1:** Pathophysiological mechanisms of AAS and potential cardiovascular consequences.

Mechanism	Pathophysiological Change	Potential Clinical Consequence
Atherogenic dyslipidemia	↑ LDL-C, ↓ HDL-C, ↑ VLDL; inhibition of apoA-I and HDL synthesis; accelerated HDL catabolism	Accelerated atherosclerosis, premature CAD, MI, sudden cardiac death
Hypertension	↑ Sympathetic tone; ↑ renin release; ↑ aldosterone → sodium/water retention, oxidative stress, fibrosis; upregulation of AT1R	Secondary hypertension, LVH, HF, vascular remodeling
Prothrombotic state	↑ Erythropoiesis (↑ EPO, ↓ hepcidin); ↑ hematocrit/viscosity; ↑ platelet TXA_2_ receptor density and aggregation; ↑ factors II, VIII, IX; ↓ fibrinolysis (↑ PAI-1, fibrinogen, FXIII)	VTE, stroke, MI, pulmonary embolism
Endothelial dysfunction	↓ NO, ↓ prostacyclin, impaired EDHF; ↑ adhesion molecules (VCAM-1, ICAM-1, E-selectin); proinflammatory endothelial phenotype	Vasoconstriction, CAD, hypertension, increased vascular stiffness
Oxidative stress and mitochondrial dysfunction	↑ ROS (H_2_O_2_, superoxide); mitochondrial Ca^2+^ dysregulation; ↓ membrane potential; lipid/protein/DNA oxidation; ↓ antioxidant enzymes; vicious cycle of ROS–mitochondria	LVH, interstitial fibrosis, diastolic dysfunction, arrhythmias
Apoptosis	AR overstimulation → cytochrome c release, caspase-3 activation, ↑ p53; TLR4/NF-κB/NLRP3 inflammasome activation → pyroptosis	Cardiomyocyte loss, myocardial thinning, remodeling, cardiomyopathy, SCD
Myocardial remodeling	AR activation → hypertrophy; TGF-β/SMAD/CTGF-driven fibroblast activation; ↑ collagen I/III deposition, ↑ MMP/TIMP imbalance	LVH, interstitial fibrosis, ↓ EF, restrictive/dilated cardiomyopathy, HF
Systemic inflammation	↑ TLR4, NF-κB, NLRP3 inflammasome; ↑ cytokines (TNF-α, IL-6, IL-1β); ↑ hs-CRP	Endothelial injury, accelerated atherogenesis, plaque instability

**Abbreviations:** AAS—Anabolic–androgenic steroids; apoA-I—Apolipoprotein A-I; AR—Androgen receptor; AT1R—Angiotensin II type 1 receptor; CAD—Coronary artery disease; CTGF—Connective tissue growth factor; EDHF—Endothelium-derived hyperpolarizing factor; EF—Ejection fraction; EPO—Erythropoietin; FXIII—Factor XIII; HF—Heart failure; hs-CRP—High-sensitivity C-reactive protein; ICAM-1—Intercellular adhesion molecule-1; IL-1β—Interleukin-1 beta; IL-6—Interleukin-6; LDL-C—Low-density lipoprotein cholesterol; LV—Left ventricle; LVH—Left ventricular hypertrophy; MI—Myocardial infarction; MMP—Matrix metalloproteinase; NF-κB—Nuclear factor kappa-light-chain-enhancer of activated B cells; NO—Nitric oxide; NLRP3—NOD-like receptor family pyrin domain containing 3; PAI-1—Plasminogen activator inhibitor type 1ROS—Reactive oxygen species; SCD—Sudden cardiac death; SMAD—Mothers against decapentaplegic homolog; TGF-β—Transforming growth factor beta; TIMP—Tissue Inhibitor of Metalloproteinases; TLR4—Toll-like receptor 4; TXA_2_—Thromboxane A_2_; VCAM-1—Vascular cell adhesion molecule-1; VLDL—Very-low-density lipoprotein; VTE—Venous thromboembolism. ↑—increase, ↓—decrease.

**Table 2:** Summary of key clinical studies on the cardiovascular effects of AAS abuse.

Study (Year)	Population/AAS Exposure	Key Cardiovascular Findings
Windfeld-Mathiasen et al. (2025) Circulation [1]	1189 male AAS users (sanctioned for doping) vs. 59,450 age-matched controls; ~11-year follow-up (Danish registry cohort)	AAS users had a markedly higher incidence of cardiovascular events vs. controls. AASs were associated with an increased risk of acute myocardial infarction (adjusted hazard ratio [aHR] 3.00 [95% CI, 1.67–5.39]), percutaneous coronary intervention or coronary artery bypass graft (aHR 2.95 [95% CI, 1.68–5.18]), venous thromboembolism (aHR 2.42 [95% CI, 1.54–3.80]), arrhythmias (aHR 2.26 [95% CI, 1.53–3.32]), cardiomyopathy (aHR 8.90 [95% CI, 4.99–15.88]), and heart failure This large study establishes AAS abuse as a significant independent risk factor for premature cardiovascular disease.
Baggish et al. (2017) Circulation [62]	Cross-sectional study of 140 male weightlifters (86 long-term AAS users with ≥2 years use vs. 54 non-users); median ~9 years of cumulative AAS exposure	AAS users showed reduced left ventricular ejection fraction (∼52% vs. 63%, *p* < 0.001) and impaired diastolic function compared to non-user. They also had significantly higher coronary artery plaque volume (median 3 mL vs. 0 mL, *p* = 0.012). Ongoing AAS users had worse cardiac function than former users of AAS, suggesting partial improvement upon cessation. Cumulative AAS dose/duration correlated positively with coronary atherosclerotic burden, implicating a dose–response relationship in AAS-induced cardiac damage.
Fyksen et al. (2025) Open Heart [42]	Prospective cohort of 32 long-term AAS-using athletes (median 16-year follow-up) and 13 non-using controls. Among users, 15 discontinued AAS during follow-up while 17 continued use.	At baseline, AAS users had greater left ventricular mass and lower ejection fraction (LVEF) than control. After 16 years, users who continued AAS had persistently enlarged LV mass and reduced LVEF, whereas those who quit AAS showed significant regression of LV hypertrophy and an improvement in LVEF (median +3% vs. –2% change in LVEF for quitters vs. continuers, *p* < 0.01). This suggests that some AAS-induced cardiac changes are partially reversible after cessation, although LVEF in former users remained slightly subnormal. No difference in coronary artery disease prevalence was observed between groups.
Tungesvik et al. (2024) Sci. Reports [8]	123 young male strength athletes (56 current AAS users vs. 67 non-user weightlifting controls)	AAS use was associated with significant endothelial dysfunction. AAS users had markedly reduced carotid artery reactivity and flow-mediated dilation compared to controls (both *p* < 0.001), indicating impaired nitric oxide–mediated vasodilation. These vascular changes provide a link to the heightened risk of acute cardiac events (e.g., myocardial infarction or sudden death) in young AAS users despite often angiographically normal coronary arteries.

## Data Availability

No new data were created or analyzed in this study. Data sharing is not applicable to this article.
